# The effect of weight-loss surgery in patients with obesity on adipose tissue mesenchymal stem cells versus circulating endothelial progenitor cells

**DOI:** 10.1038/s41366-026-02057-8

**Published:** 2026-04-06

**Authors:** Ning Yang, Mina H. Al Saeedi, Ailing Xue, Negin Mahmoudi Hamidabad, Xiangyang Zhu, Hui Tang, Kyra L. Jordan, Aleksandra Kukla, Alfonso Eirin, Amir Lerman, Lilach O. Lerman

**Affiliations:** 1 Division of Nephrology and Hypertension, Mayo Clinic, Rochester, MN USA; 2 Division of Nephrology, The First Affiliated Hospital of Dalian Medical University, Dalian, China; 3 Brookdale Hospital Medical Center, Brooklyn, NY USA; 4 Division of Cardiovascular Medicine, Mayo Clinic, Rochester, MN USA

**Keywords:** Weight-loss surgery, Adipose tissue-derived mesenchymal stem cells, Endothelial progenitor cells, Obesity, Cell biology, Metabolism

## Abstract

**Background/Objective:**

Obesity imposes dysfunction of the endogenous cellular reparative system, which may manifest as impaired adipose tissue-derived mesenchymal stem/stromal cells (AT-MSCs) function or altered characteristics of circulating endothelial progenitor cells (EPCs). However, whether both systems are abnormal in patients with obesity remains unclear. We hypothesized that human obesity induces impairment of MSCs and EPCs that would be reversed after weight-loss surgery (WLS).

**Methods:**

Abdominal adipose tissue and peripheral blood mononuclear cells were collected to harvest MSCs and EPCs, respectively, from patients with obesity (*n* = 8) before and 9–12 months after WLS. MSCs mitochondrial function and EPCs number and surface markers were compared to those collected from healthy controls (HC).

**Results:**

Patients with obesity had a higher basal body mass index compared to both HC (*P* < 0.0001) and post-WLS (*P* < 0.001). Compared to HC, MSC proliferative and differentiation capacity was preserved (*P* > 0.05), but they showed at baseline increased mitochondrial oxidative stress, and cytochrome-c release (*P* < 0.05), with reduced membrane potential and matrix density, which mostly improved after WLS. The percent of circulating CD34^+^KDR^+^CD133^+^ and CD34^+^KDR^+^ EPCs was elevated in patients with obesity (*P* < 0.05), as were EPC fractions expressing the inflammatory marker VAP-1 or pro-calcinogenic marker OCN-1, yet neither fell after WLS (*P* > 0.05).

**Conclusion:**

Obesity impairs MSC mitochondrial function and increases the percent of circulating, but also potentially injurious EPCs. WLS largely reverses MSC mitochondrial injury and but not circulating EPC characteristics. Therefore, restoration of the endogenous tissue-resident and circulating cellular regenerative systems in the same patients with obesity may require different strategies or timeframes.

## Introduction

The global obesity epidemic continues to rise. Obesity is commonly associated with endocrine and metabolic consequences that contribute to the onset of numerous health problems, including cardiovascular disease, diabetes mellitus, and hypertension [1], possibly partly due to inadequate endogenous repair processes [2]. Hence, reduction in obesity could not only decrease the risk of associated morbidities but also improve overall metabolic health and potentially extend lifespan.

The endogenous cellular regenerative system includes both tissue-resident and blood-borne circulating cells that are charged with replacing or repairing damaged tissue cells and vascular endothelial cells, respectively. Mesenchymal stem/stromal cells (MSC) are endogenous tissue resident, self-renewing cells, capable of differentiating into mature cell lineages and selectively boosting cellular regeneration [3]. Among them, adipose tissue-derived MSCs (AT-MSCs) have the advantages of easy harvesting from fat, high proliferation rate, multipotency, and robust immunomodulatory capabilities [3]. However, in previous studies we have demonstrated that obesity induces senescence and dysfunction in both porcine and human AT-MSCs [4, 5]. This may contribute to the impaired repair capacity of both endogenous MSCs in patients with obesity as well as reduce the effectiveness of exogenous autologous MSC-base therapy.

Weight-loss surgery (WLS) is considered the most effective treatment for obesity [6]. Studies have shown that WLS reshapes some AT-MSCs characteristics, including reduced DNA damage, enhanced viability, prolonged replicative lifespan, decreased adipogenic differentiation, and increased ability to dedifferentiate into preadipocytes [7, 8]. Notably, we have shown that mitochondrial damage is a key feature of AT-MSC impairment caused by obesity [9]. WLS has been shown to improve mitochondrial function in skeletal muscle cells [10] and peripheral blood monocytes [11], but whether WLS can improve obesity-induced mitochondrial damage in AT-MSCs remains unclear.

Endothelial progenitor cells (EPCs) comprise a bone marrow-derived circulating reparative system that patrols the vasculature to repair injured vessels and tissues. Some studies showed that the number of EPCs decreased in patients with obesity [12, 13], whereas others found increased numbers of EPCs that directly correlated with body mass index (BMI) in patients with obesity [14]. Similarly, the numbers of circulating EPCs were reported to increase^5^or decreased [14] after WLS. Therefore, the relationship between obesity and EPC numbers and the impact of WLS are incompletely understood. Furthermore, the effect of WLS on concurrent MSC and EPC phenotypes in the same individuals remains unknown.

Importantly, most prior studies have investigated AT-MSCs or EPCs alone, but not concurrently in the same individuals with obesity. AT-MSCs and EPCs represent complementary regenerative systems: MSCs act locally within adipose tissue, whereas EPCs circulate to repair vascular injury. Thus, a dual analysis of these systems provides a more comprehensive view of endogenous repair capacity and its modulation by obesity and WLS. This approach not only advances mechanistic understanding but also has translational implications, as it may guide optimization of autologous cell-based therapies and vascular repair strategies in patients with obesity.

Therefore, this study aimed to test the hypothesis that obesity alters and WLS would restore AT-MSCs mitochondrial function and EPC phenotype. To this end, abdominal adipose tissue and peripheral blood were collected from healthy controls (HC) and patients with obesity both before and after WLS to harvest and compare the characteristics of AT-MSCs and EPCs.

## Materials and methods

### Study subjects

Patients aged 18–80 years with a BMI > 30 kg/m² who were scheduled for WLS were recruited (*n* = 8). Exclusion criteria included pregnancy, chronic inflammatory disease, active malignancy, recent stroke or myocardial infarction, and solid organ transplantation. Patients with obesity underwent either laparoscopic sleeve gastrectomy or gastric bypass, and abdominal subcutaneous adipose tissue and PBMCs were collected during WLS and 9–12 months later using a biopsy and blood draw, respectively. Clinical and laboratory data were obtained from the pre- and post-WLS medical visit. Age- and sex-matched healthy individuals with BMI < 25 kg/m² (kidney donors undergoing kidney donation surgery, *n* = 8) were included as HC for MSC harvesting, while EPC were collected from a comparable group of HCs (*n* = 7) that were matched with the kidney donors for sex, age and BMI. All participants provided written informed consent, and the study was approved by the Mayo Clinic Institutional Review Board (IRB18-005076).

### Isolation and phenotyping of AT-MSC

AT-MSCs were isolated from similar locations of abdominal fat in all the patients. The harvested fat was cut into 2–4 mm pieces and AT-MSCs were isolated using the Adipose Tissue Dissociation Kit (Miltenyi Biotec, Cat#130-105-808, Auburn, CA, USA) following manufacturer’s protocols. The cell pellet was resuspended in Advanced Minimum Essential Medium supplemented with 5% platelet lysate (PLTmax, Mill-Creek Life Sciences, Cat#SCM141, Darmstadt, Germany). AT-MSCs were subsequently expanded through three passages to prepare for the next experiments. Expression of MSC markers (CD73+, CD90+, CD105+, CD34−, CD45−) was verified as described in our previous study [5].

### MSC functional analyses

MSC differentiation capacity was evaluated according to the manufacturer’s instructions (Human MSC Functional Identification Kit, R&D, Cat#SC006, Minneapolis, MN, USA). After 21 days in culture, MSCs phenotypes were assessed by immunofluorescent staining using anti-mouse FABP4 anti-human Aggrecan, and anti-human Osteocalcin antibodies to confirm the differentiation of MSCs into adipocytes, chondrocytes, and osteocytes, respectively [5]. The percentage of positive cells was quantified using ImageJ software (National Institutes of Health, NIH).

MSC proliferation was assessed using the Incucyte® live-cell analysis and imaging system (CYTATION-5, BioTek, Santa Clara, CA, USA). Approximately 3 × 10⁴ MSCs/well were seeded in a 24-well plate and cultured for 72 h. Cell confluence was monitored every hour over 72 hours, and the data were analyzed using Gen5 software (Bio-Tek) [5].

### MSC mitochondrial morphology and function

Mitochondria morphology was evaluated using digital transmission electron microscopy (Phillips CM10) [9]. MSCs were harvested and fixed in 4% formaldehyde and 0.1% glutaraldehyde in 0.1 M phosphate-buffered saline (PBS) at 4 °C [15]. Representative MSC images were randomly selected, and mitochondria area (nm²) and matrix density were quantified as previously described [9, 16].

### MSC mitochondrial function

Mitochondrial reactive oxygen species (ROS) production was assessed using MitoSOX staining (Invitrogen, Cat#M36005, Waltham, MA, USA) and membrane potential with tetramethylrhodamine ethyl-ester (TMRE, Invitrogen, Cat#T668). The area of red fluorescence was quantified using ImageJ [9, 16].

In addition, mitochondrial function was studied in isolated mitochondria. MSCs were washed with ice-cold PBS, and 1 mL of 1× mitochondria isolation buffer was then added, the suspension centrifuged, and the pellet discarded. The supernatant was centrifuged, and the pellet was collected for isolated mitochondria.

MSC cellular and mitochondria proteins were extracted using RIPA buffer and quantified using the BCA assay. Equal amounts of protein were separated on a 4–12% SDS-PAGE gel and transferred to a PVDF membrane. After blocking with 5% non-fat dry milk in TBS-T, membranes were incubated with cytochrome-c antibody (Thermofisher, Cat#33-8500, Waltham, MA, USA), SLC25A4 (CST, Cat#51755, Danvers, MA, USA) and PGC-1α (Abcam, Cat#54481, Waltham, MA, USA) overnight at 4 °C, followed by HRP-conjugated secondary antibodies for 1 h at room temperature. Protein bands were detected using SuperSignal West Atto ultimate sensitivity (Thermofisher, Cat#YJ386614) or West Pico Plus chemiluminescent substrate (Thermofisher, Cat# ZB384075) and quantified with ImageJ, normalized to loading controls (COX-IV for mitochondria and GAPDH for cells). The ratio of mitochondrial to cellular cytochrome-c expression was calculated [17]. In addition, ATP/ADP ratio (Sigma, Cat#MAK135, Darmstadt, Germany), H₂O₂ (Abcam, Cat#102500), and COX IV activity (Abcam, Cat#109909) in whole MSCs and mitochondria were measured using kits according to the manufacturer’s instructions.

### Isolation and characterization of EPC

PBMCs were isolated using the Histopaque method and then frozen at −80 °C. For acquisition, 1 × 10^6^ cells per sample were analyzed using a FlowSight® Imaging Flow Cytometer (Amnis, Seattle, WA), with data collected using Amnis® INSPIRE® software (Version 6.0). (Supplementary Figs. S1, S2). Data analysis was performed using Amnis® IDEAS® software (Amnis Corporation, Seattle, WA) Version 6.2.

### Statistical analysis

Statistical analyses were performed using Prism (GraphPad, version 10). Normally distributed data are presented as mean ± standard deviation, and non-normally distributed data as median (interquartile range). One-way ANOVA (Kruskal–Wallis test) was used for comparisons among HC and patients with obesity at both Visit-1 (baseline) and Visit-2 (post-WLS). Paired *t*-test was used to compare patients with obesity at Visit-1 and Visit-2, and unpaired *t*-test to compare HC with either patients with obesity at Visit-1 or Visit-2. Homogeneity of variances was assessed using Bartlett’s or Brown–Forsythe test before performing parametric analyses. *P*-value < 0.05 was considered statistically significant.

## Results

Patients with obesity had a higher baseline BMI compared to HC (*P* < 0.0001) which fell at Visit-2 compared to Visit-1 (*P* < 0.001, Table 1), although it remained higher than HC. No other clinical or laboratory parameters were significantly different among the groups (*P* > 0.05, Table 1). Similarly, no differences were observed in age, sex, BMI, or other clinical parameters between the two HC groups used for MSC and EPC (*P* > 0.05, Supplementary Table. 1).

**Table 1:** Characteristics of enrolled patients with obesity or healthy controls (HC).

	HC	Obesity (Visit-1)	Obesity (Visit-2)
Demographics			
Age (year)	45.3 ± 11.7	48.6 ± 10.2	48.6 ± 10.2
Sex (male %)	50	62.5	62.5
Body Mass Index (kg/m^2^)	25.5 ± 2.2	42.4 ± 3.1^+^	31.1 ± 4.6^+^*
Hypertension	0/8	3/8	3/8
Diabetes	0/8	0/8	0/8
Dyslipidemia	0/8	1/8	1/8
SBP (mmHg)	110 ± 7.7	124 ± 14.4	119 ± 13.0
DBP (mmHg)	69.5 ± 9.5	74.9 ± 6.2	74.3 ± 12.6
MAP (mmHg)	83 ± 8.4	91 ± 8.1	89 ± 12.4
Type of WLS	-	1 robotic-assisted gastric bypass 4 laparoscopic gastric bypass 3 laparoscopic sleeve gastrectomy	-
Laboratory data			
Hemoglobin (g/dL)	13.8 ± 1.5	14.0 ± 1.5	13.8 ± 1.3
Serum Albumin (g/dL)	4.5 ± 0.2	4.4 ± 0.3	4.4 ± 0.4
FBG (mg/dL)	97.8 ± 2.9	94 ± 17.9	95.8 ± 13.7
Serum Creatinine (mg/dL)	0.9 ± 0.2	0.9 ± 0.2	0.8 ± 0.1
eGFR (mL/min/1.73/m^2^)	92.6 ± 14.1	92.9 ± 21.6	106.3 ± 6.9
BUN (mg/dL)	12.3 ± 3.1	14.0 ± 4.1	12.3 ± 3.5
Medication use (*n*/%)			
Antihypertension drugs	0/0	0/0	0/0
Lipid-lowering drugs	0/0	0/0	0/0

*SBP* Systolic Blood Pressure, *DBP* Diastolic Blood Pressure, *MAP* Mean Arterial Pressure, *WLS* weight-loss surgery, *FPG* Fasting Blood Glucose, *eGFR* Estimated Glomerular Filtration Rate, *BUN* Blood Urea Nitrogen.

^+^*P* < 0.0001, patients with obesity at Visit-1 and 2 vs HC; *P < 0.001, Visit-1 vs Visit-2.

### AT-MSCs from patients with obesity showed preserved differentiation and proliferation capacity

**Fig. 1:**
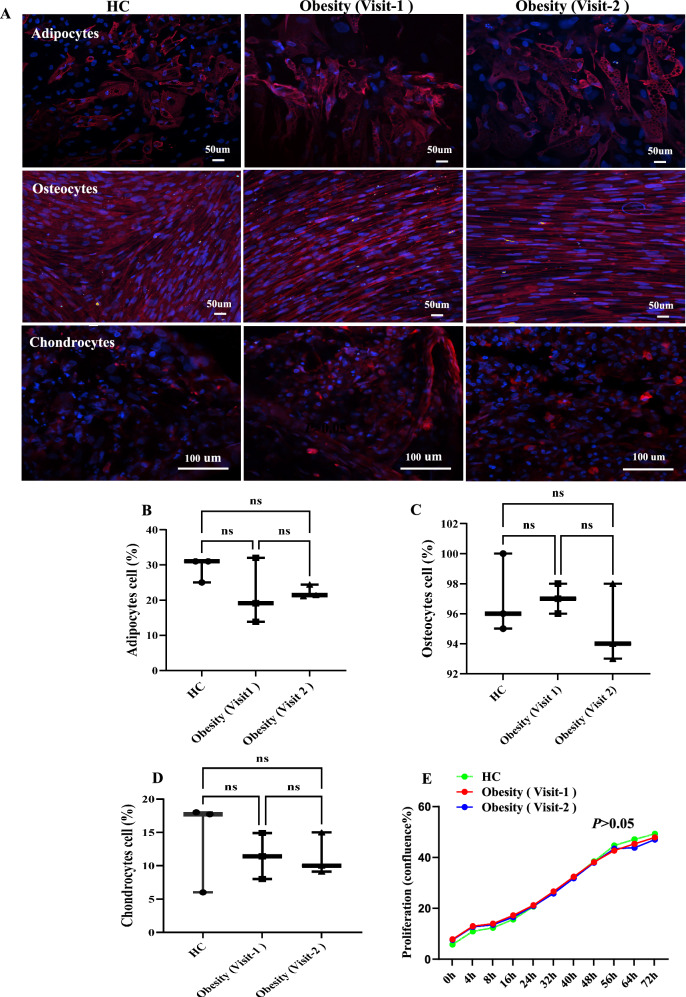
Differentiation and proliferation of adipose-derived MSCs in patients with obesity and weight-loss surgery. **A** Representative images showing differentiation of MSCs from healthy controls (HC), and patients with obesity before (Visit-1) and 9–12 months after (Visit-2) weight-loss surgery. Differentiation of adipose-derived MSCs was preserved in patients with obesity and weight-loss surgery, as indicated by a similar proportion of cells differentiating into adipocytes (**B**) osteocytes (**C**) and chondrocytes (**D**) (red) (*n* = 3/group). Their proliferation capacity was also preserved (**E**) (*n* = 8/group). **P < 0.01, patients with obesity at Visit-1 vs. Visit-2; ***P < 0.001, patients with obesity at Visit-1 vs. HC (One-way ANOVA, Kruskal–Wallis).

MSCs phenotype was confirmed through trans-differentiation into adipocytes, osteocytes, and chondrocytes. Their differentiation and proliferation capacity were similar among Visit-1, Visit-2, and HC (*P* > 0.05, Fig. 1A, E). Therefore, in our cohort, obesity did not affect the differentiation and proliferation capacity of AT-MSCs. MSCs also expressed typical MSC markers (CD73+, CD90+, CD105+, CD34−, CD45−) (not shown), confirming their identity.

### WLS partially mitigates obesity-induced AT-MSCs mitochondrial damage

**Fig. 2:**
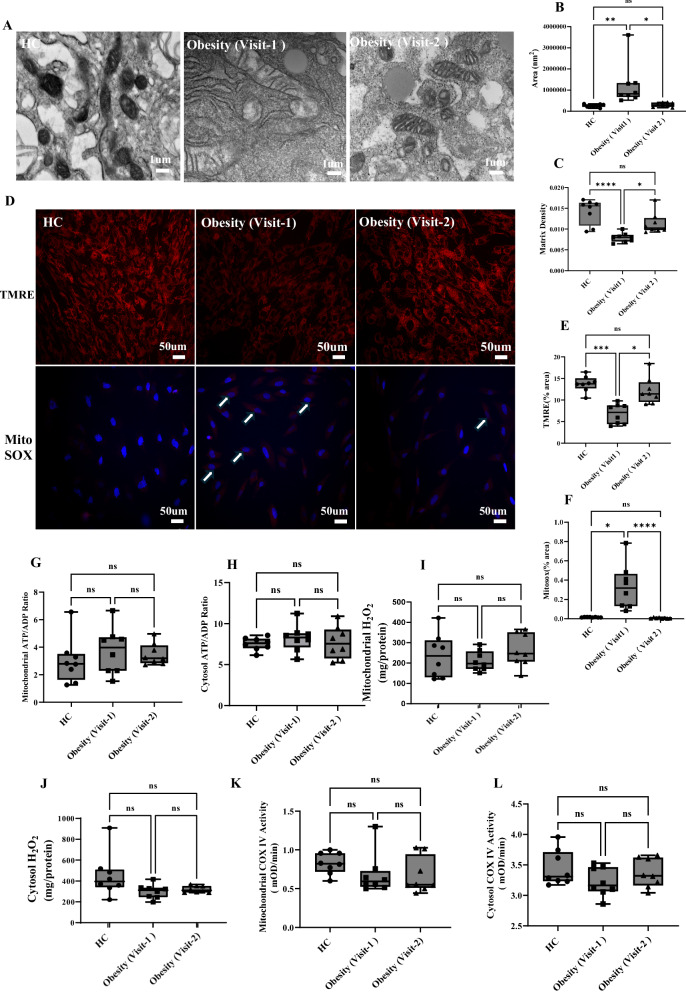
Mitochondrial function of AT-MSCs from patients with obesity improves after WLS. **A** Representative transmission electron microscopy images of AT-MSCs collected from healthy controls (HC) and patients with obesity before (Visit-1) and 9–12 months after weight-loss surgery (Visit-2) and quantification of mitochondria area (**B**) and matrix density (**C**) (*n* = 8/group). Representative Tetramethylrhodamine ethyl-ester (TMRE) and MitoSOX staining (white arrows, **D**) and their quantification (**E**, **F**) (*n* = 8/group) indicated impaired mitochondrial function of AT-MSCs in patients with obesity, which improved after WSL. On the other hand, ATP/ADP ratio, H2O2 and COX IV activity in whole MSCs and mitochondria showed no differences between HC and patients with obesity before or after WLS (**G**–**L**) (*n* = 8/group). *P < 0.05, patients with obesity at Visit-1 vs. Visit-2; **P < 0.01, ****P < 0.001, patients with obesity at Visit-1 vs. HC (One-way ANOVA, Kruskal–Wallis).

Electron microscopy showed that mitochondrial area in AT-MSCs of patients with obesity at Visit-1 was markedly increased (Visit-1 vs. HC *P* = 0.008; Visit-1 vs. Visit-2 *P* = 0.01, Fig. 2A, B) and matrix density decreased compared to both HC and Visit-2 (*P*<0.0001 and *P* = 0.02, respectively; Fig. 2A–C). Compared to HC and Visit-2, Visit-1 TMRE staining demonstrated decreased membrane potential (*P* = 0.0005 and *P* = 0.02, respectively Fig. 2D, E) and Mito SOX revealed increased oxidative stress (*P* = 0.04 and *P*˂0.0001, respectively, Fig. 2D, F). At Visit-2 After WLS, these indicators of mitochondrial injury were reduced compared to Visit-1 (TMRE *P* = 0.02; Mito SOX *P*<0.0001) suggesting improvement of mitochondrial function in obesity MSCs. Contrarily, the ATP/ADP ratio, H_2_O_2_, and COX IV activity in MSCs and mitochondria showed no significant differences between HC and patients with obesity either before or after WLS (*P* > 0.05, Fig. 2G–L). This could signify a compensatory adaptation in which MSC mitochondrial quality is maintained or restored without necessarily a corresponding increase in energetic flux, indicating that mitochondrial injury in obesity was mild. Indeed, a post-hoc power analysis using the observed differences between HC and patients with obesity at Visit-1 showed that we at our sample size were underpowered to detect differences in the ATP/ADP ratio, H_2_O_2_, and COX IV activity (statistical power of 17%, 39%, and 35%, respectively) at α = 0.05, two-sided.

**Fig. 3:**
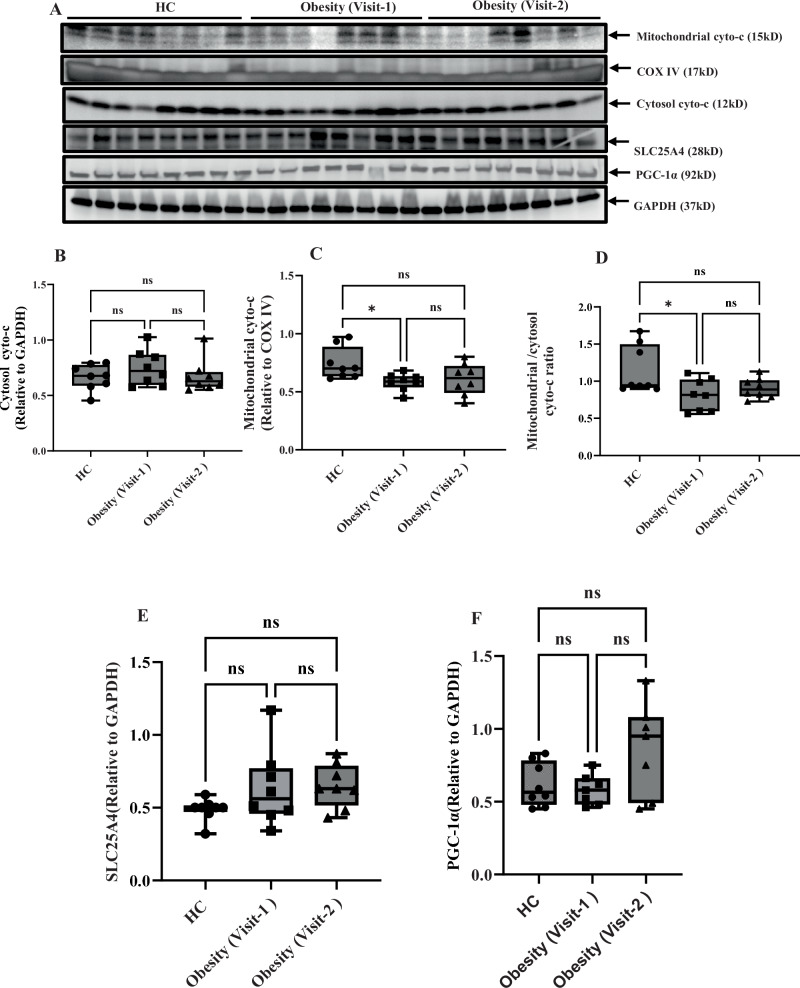
MSC and mitochondrial protein expression in obesity. Western blotting bands of mitochondrial and cytosol cytochrome-c (cyto-c), SLC25A4, and PGC-1α in AT-MSCs from healthy controls (HC) and patients with obesity before (Visit-1) and 9–12 months after weight-loss surgery (Visit-2) (**A**). Quantification showed reduced mitochondrial cytochrome-c in obesity at Visit-1 vs HC (**B**) whereas cytosol cytochrome-c (**C**) was unchanged, resulting in reduced mitochondria/cytosol cytochrome-c ratio (**D**) (*n* = 8/group). There were no differences or changes in SLC25A4 and PGC-1α expression (**E**, **F**) (*n* = 8/group). *P < 0.05, HC vs patients with obesity at Visit-1 (One-way ANOVA, Kruskal–Wallis).

We further assessed cytochrome-c release from mitochondria using western blotting. Compared to HC, MSCs from patients with obesity at Visit-1 showed a decrease in mitochondrial cytochrome-c expression and its ratio of total cytochrome-c (*P* = 0.02, Fig. 3), which after WLS were no longer significantly different from either HC or baseline (*P* > 0.05, Fig. 3). Therefore, WLS only partially restored AT-MSCs pro-apoptotic properties. Contrarily, SLC25A4 and PGC-1α expression in MSC (Fig. 3E, F) showed no significant differences between HC and patients with obesity either before or after WLS.

### Circulating EPCs characteristics were altered in patients with obesity, and partially improved by WLS

**Fig. 4:**
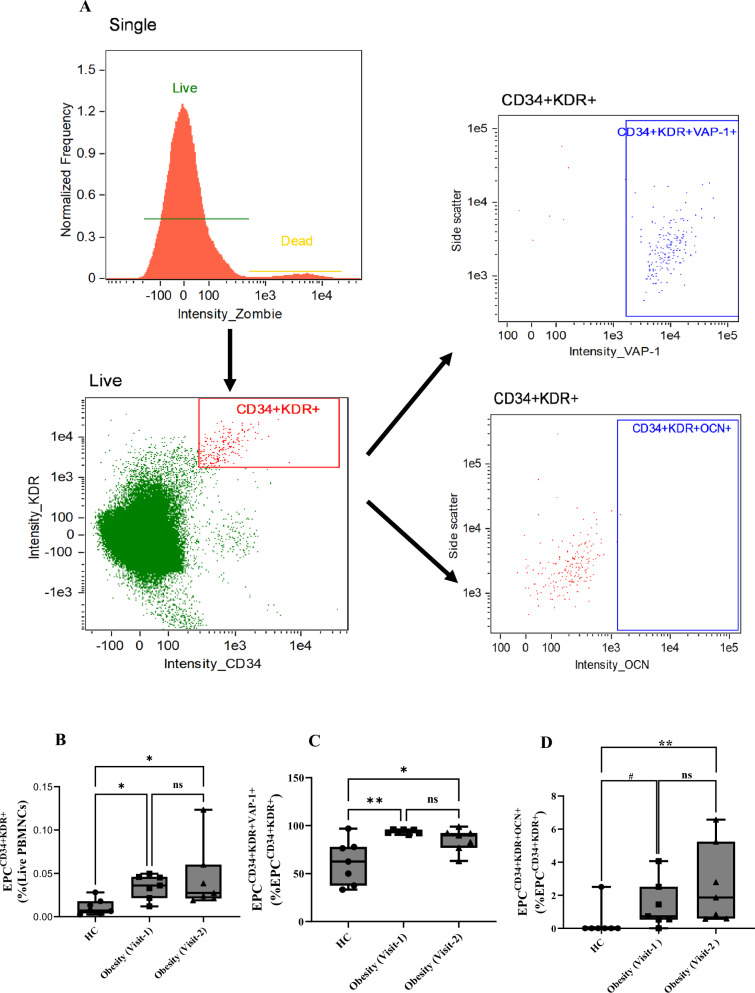
The proportion of circulating EPCs increased in patients with obesity vs. healthy controls (HC). Gating strategy for EPC (**A**). The fraction of EPCCD34+KDR+ out of PBMCs was elevated in Visit-1 and remained unchanged in Visit-2 (**B**) as was the ratio of EPCCD34+KDR+VAP-1+ (**C**) and EPCCD34+KDR+OCN+ (**D**) out of EPCCD34+KDR+ (*n* = 7/group). *P < 0.05, **P < 0.01, HC vs patients with obesity at Visit-1 and Visit-2 (One-way ANOVA, Kruskal–Wallis); # *P* <0.05, HC vs patients with obesity at Visit-1 (unpaired *t*-test).

Compared to HC, the percent of circulating mature EPCs^CD34+KDR+^ out of live PBMCs in patients with obesity at Visit-1 was significantly increased (*P* = 0.02, Fig. 4A, B), as were the percents of EPCs^CD34+KDR+VAP-1+^ (*P* = 0.002, Fig. 4C) and EPCs^CD34+KDR+ OCN+^ (*P* = 0.02, Fig. 4D) out of EPCs^CD34+KDR+^ and both remained unchanged at Visit-2.

**Fig. 5:**
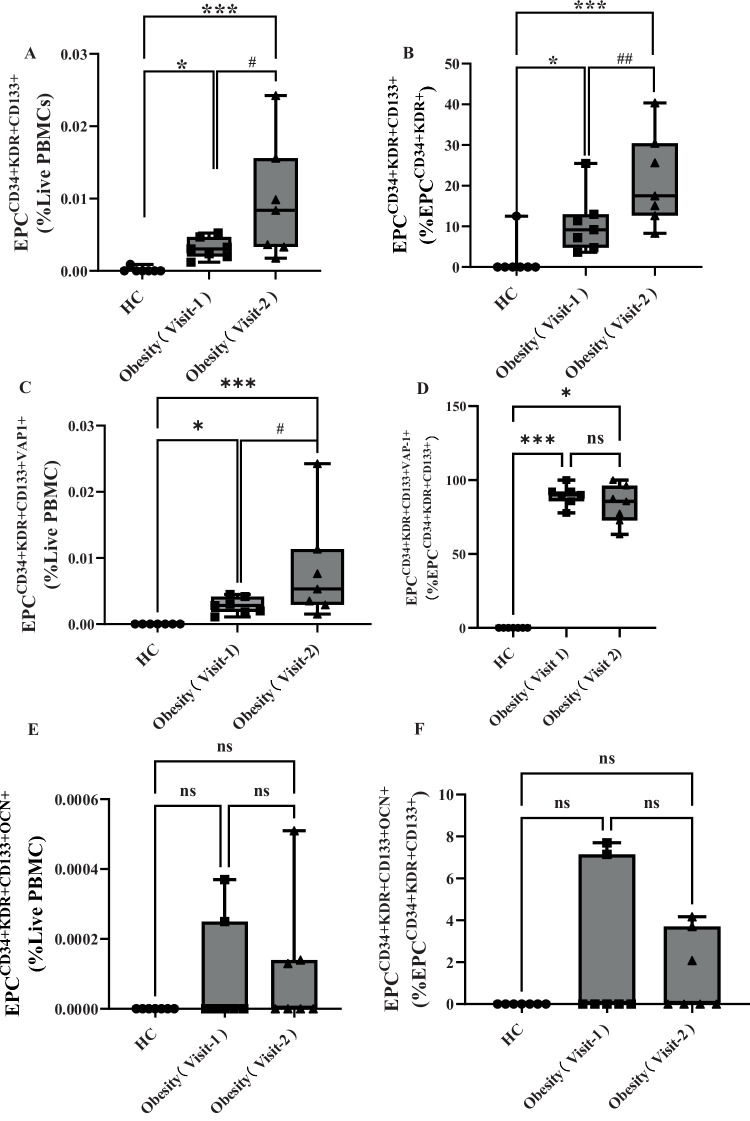
The proportions of circulating EPCCD34+KDR+CD133+ and EPCCD34+KDR+C133+VAP-1+ were increased in patients with obesity vs. healthy controls (HC). Compared with HC, the fraction of EPCCD34+KDR+CD133+ out of live PBMCs and of EPCCD34+KDR+ increased at both Visit-1 and Visit-2 (**A**, **B**) as was the ratio of EPCCD34+KDR+CD133+VAP-1+ out of both PBMC and EPCCD34+KDR+CD133+ (**C**, **D**). After WLS, compared with Visit-1, the fraction of EPCCD34+KDR+CD133+ out of both PBMCs and EPCCD34+KDR+ and EPCCD34+KDR+CD133+VAP-1+ out of PBMCs were increased, but not out of EPCCD34+KDR+CD133+ (**D**). However, the percentage of EPCsCD34+KDR+CD133+OCN+ out of PBMCs and EPCsCD34+KDR+CD133+ showed no significant difference among these groups (**E**, **F**) (*n* = 7/group). *P < 0.05, ***P < 0.001, HC vs patients with obesity at Visit-1 and Visit-2 (One-way ANOVA, Kruskal–Wallis); #*P* < 0.05, ##*P* < 0.01, patients with obesity Visit-1 vs Visit-2 (paired *t*-test).

Similarly, the percentage of circulating EPCs^CD34+KDR+CD133+^ out of both PBMCs and EPCs^CD34+KDR+^ from patients with obesity at Visit-1 was significantly elevated compared to HC (*P* = 0.03, Fig. 5A, B). Interestingly, both fractions increased or tended to increase further after WLS (*P* = 0.03 and *P* = 0.075, respectively, Fig. 5A, B). Moreover, the percentage of circulating EPCs^CD34+KDR+CD133+VAP-1+^ out of both PBMCs (*P* = 0.02, Fig. 5C) and of EPCs^CD34+KDR+CD133+^ (*P* = 0.0009, Fig. 5D) from patients with obesity at Visit-1 were also significantly increased compared to HC. The percentage of circulating EPCs^CD34+KDR+CD133+VAP-1+^ out of PBMCs significantly increased after WLS (*P* = 0.03, Fig. 5C), but not their fractions out of EPCs^CD34+KDR+CD133+^ (*P* ˃ 0.05, Fig. 5D). On the other hand, no significant differences were observed in OCN+ immature EPCs (EPCs^CD34+KDR+CD133+OCN+^ out of PBMCs or EPCs^CD34+KDR+CD133+^) among these groups (*P* ˃ 0.05, Fig. 5E, F), although their fraction of OCN+ out of mature EPCs were elevated at both time points. Hence, obesity increases the fractions of both mature (EPCs^CD34+KDR+^) and immature (EPCs^CD34+KDR+CD133+^) circulating EPCs and imposes pro-inflammatory (VAP-1+) and pro-calcinogenic (OCN+) properties, which are not mitigated within a year after WLS. However, there were no statistically significant correlations between plasma IL-6 and EPCs^CD34+KDR+OCN+^ or EPCs^CD34+KDR+VAP-1+^ (Supplementary Fig. S3).

## Discussion

Our study demonstrates that obesity induces in the same patients marked MSC mitochondrial damage accompanied by EPC phenotypic alterations. Obesity increased the percent of circulating immature (CD34+KDR+CD133+) and mature (CD34+KDR+) EPCs, as well as those with pro-inflammatory (VAP-1+) and pro-calcinogenic (OCN+) properties. Notably, WLS attenuated MSC mitochondrial injury, but did not affect or even further magnified EPC alterations. Hence, weight-loss achieves in the same patients with obesity incomplete reinstatement of the endogenous reparatory system, with greater improvement in the tissue-resident than in the circulating cellular repair systems. These observations suggest that different strategies or timeframes may be required to restore these cellular regenerative systems in obesity and underscore a need for additional studies to understand and reverse the underlying mechanisms.

Obesity is defined by adipose tissue inflammation and remodeling and dysregulated cytokine secretion [18]. Recently, there has been increasing focus on the morphological and functional changes induced by obesity in tissue-resident MSCs, with altered proliferative and differentiation capacity, impaired cell-cycle progression, and accelerated cellular senescence [5, 19]. Obesity-induced adipocyte enlargement creates a hypoxic environment with delayed neovascularization [20] and impaired immune cell recruitment [21], all of which compromise the MSC-conferred tissue repair and regenerative functions [5, 9]. This results in abnormal healing processes in patients with obesity, like delayed wound healing [22, 23].

WLS is considered an effective treatment for severe obesity, achieving sustained long-term weight-loss and reducing obesity-related complications [24], and may also change the characteristics of MSCs and EPCs derived from patients with obesity. WLS partially restored the function of AT-MSCs in adipogenesis and cytokine secretion [25], and reprogramed AT-MSCs, with reduced DNA-damage, improved viability, extended replicative lifespan, and reduced adipogenic differentiation potential [7]. A recent study demonstrated higher activity of AT-MSCs from females and younger individuals who lost weight through increased exercise and dietary changes [26]. Nevertheless, even after weight-loss AT-MSCs purportedly retain an adipogenic memory [10], and may show an augmented adipogenic potential [27].

Mitochondrial dysfunction characterizes human obesity [28], which may be reflected in mitophagy and epigenetic changes in mitochondria-related genes, and accompanied by impaired fatty acid metabolism and neurogenic differentiation capacity [9, 29]. We observed that AT-MSCs from patients with obesity exhibit some aspects of mitochondrial damage, including decreased mitochondrial matrix density and membrane potential and increased superoxide production, consistent with our previous findings [9, 17]. Importantly, mitochondrial damage was partially improved by weight-loss through WSL (except for cytochrome-c release), which might be associated with improved repair capacity. Our findings are underscored by an earlier study showing that AT-MSC from mice that lost weight through dietary control exhibited a higher number of mitochondria and lower ROS production and oxygen consumption [30]. Cytochrome-c translocation from the mitochondria to the cellular cytosol is a key step in the process of cellular apoptosis [31]. Hence, we measured the ratio of mitochondrial and cytosol cytochrome-c expression. While this is an imprecise index of their relative distributions, we found it decreased in MSCs from patients with obesity, consistent with pro-apoptotic activity. This may be secondary to the obesity-induced decrease in mitochondrial membrane potential and increased ROS [32], although cytochrome-c release did not significantly improve after WLS. On the other hand, other aspects of mitochondrial damage, such as ATP production, H_2_O_2_, COX IV activity, and SLC25A4 and PGC-1α expression were preserved in MSC from patients with obesity, indicating that mitochondrial injury in obesity was relatively mild. Nevertheless, our study was underpowered to detect ATP/ADP and H_2_O_2_ and these assays may be insensitive to subtle mitochondrial function alterations, particularly in heterogeneous clinical samples and when assessed in vitro.

EPCs are mobilized from the bone marrow into peripheral blood, mediating endothelial repair in response to injury [33] and like MSCs are also modulated by obesity. Early studies found EPCs numbers and function along with premature senescence in patients with obesity [34, 35], whereas others found that the number of EPCs in patients with obesity [36] was contrarily increased [14]. Therefore, the relationship between obesity and EPCs remained to be elucidated.

EPCs comprise distinct subpopulations. Among them, EPCs^CD34⁺KDR⁺CD133⁺^ mobilized from bone marrow were considered immature. As EPCs enter the peripheral blood, they gradually lose CD133 expression, with EPCs^CD34⁺KDR⁺^ subsequently becoming the predominant circulating EPC population [37, 38]. Our finding showed that the percent of both immature and mature circulating EPCs were significantly elevated in patients with obesity.

The reported effects of obesity and WLS on EPC numbers have been inconsistent. The numbers of EPC-like outgrowth cells cultured in vitro were lower in normotensive and hypertensive patients with obesity and increased after WLS [5], whereas circulating CD34^+^/KDR^+^/CD45^-^EPC numbers were elevated in obesity and fell after WLS [7]. Another study found a fall in CD34^+^/KDR^+^/CD133^+^ EPC in older patients with obesity, who also showed higher blood pressure and insulin resistance compared to controls [39]. Likely, the duration of obesity and comorbid conditions affect EPC numbers. Moreover, a discrepancy in EPCs enumeration with a reported fall in patients with obesity [35, 36] may be due to differences in detection methods and definition. For example, in vitro EPC assays (marked by UEA-1+ acLDL-DiI+) assess functional EPCs, whereas flow cytometry identifies circulating EPCs by their surface markers, reflecting mobilization dynamics. Interestingly, after WLS the proportion of circulating immature EPCs in our patients further increased, possibly due to continued mobilization from the bone-marrow, with no change in mature EPCs. The bone-marrow microenvironment that maintains hematopoietic stem cells becomes dysregulated in cardiometabolic diseases, potentially persisting after weight loss [40]. A previous study reporting unchanged number of EPCs defined by CD34^+^KDR^+^ after weight loss in patients with obesity [6] is consistent with our observations. Contrarily, elevated EPC numbers may reflect enhanced mobilization from the bone-marrow or increased vascular turnover and remodeling in response to metabolic improvements post-WLS. Further studies integrating several methodologies are needed to fully understand the impact of WLS on EPC biology.

Importantly, in the same patients with obesity we also found increase proportions of EPCs with potentially deleterious properties. OCN is an osteoblast-derived hormone implicated in inducing calcifications, and increased OCN⁺EPCs in our patients may signify increased cardiovascular risk [41]. VAP-1 is expressed in the vascular system, especially on the surface of endothelial cells, and regulates the adhesion and migration of circulating immune cells [42]. VAP-1 is also released by adipose cells and contributes to atherogenesis in obesity [43]. We have previously shown elevated numbers of circulating inflammatory endothelial cells in blacks with essential hypertension [44] and the current study shows not only that EPC can also express VAP-1, but also that their proportion is increased in patients with obesity. These observations suggest that while EPC number rises in obesity, at least a fraction includes proinflammatory rather than reparative EPCs. Indeed, while we found no significant correlation between plasma IL-6 and either EPCs^CD34+KDR+OCN+^ or EPCs^CD34+KDR+VAP-1+^, these findings do not preclude a link to other forms of vascular injury.

The increased proportion of immature EPCs and their pro-inflammatory properties (VAP-1^+^) further increased after WLS may be related to some lingering obesity after WLS, or it might take longer for bone-marrow-derived cells to improve after weight loss [40]. The differential response of AT-MSCs and EPCs to WLS likely reflects fundamental differences in cellular biology, microenvironmental regulation, and functional roles. MSCs are tissue-resident cells within adipose depots, where local cues, like extracellular matrix composition, paracrine signaling, and oxygen tension, directly modulate mitochondrial function and regenerative capacity [45]. The local subcutaneous fat microenvironment after weight-loss may revert to a less-inflammatory state that promotes restoration of MSC function [46, 47]. In contrast, circulating bone-marrow-derived EPCs are continuously exposed to systemic factors, including inflammatory cytokines, metabolic stress, and hemodynamic forces, which may sustain their activation or dysregulation even after weight-loss surgery [38, 48]. This may account for the differences in the recovery ability between MSCs and EPCs after WLS.

MSCs and EPCs alterations might adversely impact endogenous repair in obesity. Our previous studies found that human obesity impairs the reparative properties of AT-MSCs for both in vivo and in vitro [5, 49]. Obesity-induced concurrent alterations in the characteristics of both MSCs and EPCs might thus interfere with both vascular and tissue repair. Interestingly, weight reduction more effectively restored MSCs function, providing a means for endogenous tissue repair, whereas endothelial repair may take longer time to restore. Elucidating the concomitant impact of obesity on the two cellular reparative systems may allow development of targeted strategies to restore them. Similarly, understanding the reversibility of their behavior in obesity after weight-loss is crucial for their potential application in regenerative medicine.

This study has several limitations. For example, the small number of patients reduces statistical power to detect small-to-moderate functional differences between groups or subgroups (e.g., surgery method). Because our cohorts were biologically heterogeneous and MSC/EPC readouts varied among individuals, the sample size primarily allowed detection of large effects; negative results for metrics like mitochondrial function should therefore be interpreted cautiously. Therefore, our study should be considered exploratory and hypothesis-generating. In addition, after WLS, the patients remained slightly overweight, which might have impacted the reversibility of MSCs and EPCs damage. In a previous study we found decreased proliferative capacity of MSCs in patients with obesity [4]. However, our current cohort was smaller and the patients were younger, which may have hindered our ability to detect subtle changes in MSC function [29]. Also, EPCs were identified only based on surface markers (which may also be expressed by other circulating cells), as the small numbers of circulating EPCs that can be collected limit the feasibility of functional studies. This, they should be considered “EPC-like or putative EPCs”. Additionally, because fat tissue and blood samples were not consistently collected from the same donors, MSC and EPC samples were obtained from two separate HC cohorts. Although kidney donors are generally healthy, and the two cohorts were matched for sex, age, and BMI to minimize potential confounding, this design may still introduce variability and limit statistical power compared with the use of a single, matched control cohort. Finally, additional sensitive assays of MSCs and EPCs mitochondrial function are needed to elucidate the effects of obesity on these organelles. Future studies with larger sample sizes and longitudinal designs are warranted to validate and extend our findings.

In summary, we provide important insights into the propensity of obesity to impair concurrently MSC and EPC function. This dual impairment might impose an important hindrance on the capacity for coordinated endogenous endothelial and tissue repair in obesity as well as the utility of applying exogenous autologous cell-based therapy. The ability of WLS to improve MSC, but not EPC characteristics in the same cohort might warrant alternative approaches to address vascular damage in obesity. Further, future studies with larger cohorts and longitudinal follow-ups are needed to confirm these results and explore the underlying molecular mechanisms. These findings may increase the understanding of MSC and EPC biology in obesity and close knowledge gaps that limit the advancement of regeneration medicine in patients with obesity.

## Supplementary information

Supplementary Table 1

Supplementary Material

## Data Availability

All data analyzed in this study are included in this published article and its supplementary files.
