# Supplementary material for: The effect of weight-loss surgery in patients with obesity on adipose tissue mesenchymal stem cells versus circulating endothelial progenitor cells
Source: Int J Obes (Lond). 2026 Apr 6;50(7):1459–70. doi: 10.1038/s41366-026-02057-8 (PMC13391379; doi:10.1038/s41366-026-02057-8)
Supplement: Supplementary file 1 — Supplementary Table 1 [file 41366_2026_2057_MOESM1_ESM.docx]

Supplementary Table 1. Characteristics of the two groups of enrolled healthy controls (HC)

|  | HC (MSC)  n= 8 | HC (EPC)  n= 7 |
| --- | --- | --- |
| Demographics |  |  |
| Age (year) | 45.3±11.7 | 48.6±10.2 |
| Sex (male %) | 50 | 28.5 |
| BMI (kg/m^2^) | 25.5±2.2 | 25.12±7.7 |
| Hypertension | 0/8 | 0/7 |
| Diabetes | 0/8 | 0/7 |
| Dyslipidemia | 0/8 | 0/7 |
| SBP (mmHg) | 110±7.7 | 109±14.0 |
| DBP (mmHg) | 69.5±9.5 | 62.1±13.5 |
| Laboratory data |  |  |
| Hemoglobin (g/dL） | 13.8±1.5 | 13.9±1.4 |
| Serum Albumin (g/dL) | 4.4±0.2 | 4.5±0.2 |
| FBG (mg/dL) | 97.8±2.9 | 102.5±13.8 |
| Serum Creatinine (mg/dL) | 0.9±0.2 | 1.0±0.4 |
| eGFR (mL/min/1.73/m^2^) | 92.6±14.1 | 79.3±18.7 |
| BUN (mg/dL) | 12.3±3.1 | 18.5±8.8 |

MSC: Mesenchymal stem/stromal cells; EPC: Endothelial progenitor cells; BMI: Body Mass Index; SBP: Systolic Blood Pressure; DBP: Diastolic Blood Pressure; MAP: Mean Arterial Pressure; FPG: Fasting Blood Glucose; eGFR: Estimated Glomerular Filtration Rate; CKD-EPI: Chronic Kidney Disease Epidemiology Collaboration equation; BUN: Blood Urea Nitrogen.
