# Supplementary material for: The effect of weight-loss surgery in patients with obesity on adipose tissue mesenchymal stem cells versus circulating endothelial progenitor cells
Source: Int J Obes (Lond). 2026 Apr 6;50(7):1459–70. doi: 10.1038/s41366-026-02057-8 (PMC13391379; doi:10.1038/s41366-026-02057-8)
Supplement: Supplementary file 2 — Supplementary Material [file 41366_2026_2057_MOESM2_ESM.pdf]

The Effect of Weight-loss surgery in Patients with Obesity on Adipose Tissue Mesenchymal Stem Cells versus Circulating Endothelial Progenitor Cells

Supplementary information

Supplementary methods

Single cells were gated by scatterplot of brightfield area and aspect ratio from focused cells. Subsequently, live cells were gated by histogram of Zombie live/dead negative population of single cells. Using visual confirmation, the appropriate gates were established and adjusted according to the positively stained PBMNCs in scatterplot as well as unstained PBMNCs for negative thresholding. This information was used to determine baseline thresholds to use for running PBMNCs samples. We used the thresholds generated by stained and unstained PBMNCs, and made refined the adjustments based on what the visualized in the images of the PBMNCs images in the scatterplot, this which allowed us to carefully determine the threshold at which the PBMNCs were positively stained. Invitrogen™ ABC™ Total Antibody Compensation Beads were used to generate consistent and accurate compensation matrices correcting for overlapping emissions in each of the fluorescent channels used. Using the live cells population, we gated the positive populations based on the intensity generated in the scatterplot from their respective channels.PBMCs were stained with the antibodies PerCP anti-mouse CD34 (BD, Cat#340666, Franklin Lakes, New Jersey, USA), APC anti-mouse KDR (Sino-Biological, Cat#10012-MM06- A, Wayne, PA, USA), FITC VAP-1 anti-rabbit (ThermoFisher, Cat#NB014709) and PE anti- human OCN (BD, Cat#564146).$CD34^+/KDR^+$ cells were gated from live single-cells using dot plots. Subsequently, $CD34^+/KDR^+/CD133^+$ cells were gated from the $CD34^+/KDR^+$ population by their expression[1]. Next, $VAP\text{-}1^+$ and OCN^+ cells were gated from $CD34^+/KDR^+$ and $CD34^+/KDR^+/CD133^+$ based on their expression. EPC fractions were expressed as a percentage of live PBMC and $VAP\text{-}1^+$ EPCs and OCN^+ EPC as a percentage of $CD34^+/KDR^+$ and $CD34^+/KDR^+/CD133^+$, respectively[2,3].

References

1.Fadini GP, Coracina A, Baesso I, Agostini C, Tiengo A, Avogaro A, de Kreutzenberg SV: Peripheral blood CD34+KDR+ endothelial progenitor cells are determinants of subclinical atherosclerosis in a middle-aged general population. Stroke 2006, 37:2277–82.

2.Eirin A, Zhu XY, Woollard JR, Herrmann SM, Gloviczki ML, Saad A, Juncos LA, Calhoun DA, Rule AD, Lerman A, Textor SC, Lerman LO: Increased circulating inflammatory endothelial cells in blacks with essential hypertension. Hypertension 2013, 62:585–91.

3.Ozcan I, Kanaji Y, Rajotia A, Toya T, Akhiyat N, Morse D, Lerman LO, Lerman A: Fraction of Osteocalcin Endothelial Progenitor Cells and Cardiovascular Risk. Circ Res 2023, 132:1162–4.

Supplementary figure legends

Figure S1.Unstained cells were used to set the negative gate.A.$CD34^+$; B.$CD133^+$; C.KDR^+; D.OCN^+; E.VAP-1^+

Figure S2.Positively stained sample was used to define the positive gate. A.$CD34^+$; B.$CD133^+$; C.KDR^+; D.OCN^+; E.VAP-1^+

Figure S3.Plasma Il-6 were not correlated with $EPCs^{CD34+KDR+OCN+}$ (A) or $EPCs^{CD34+KDR+VAP\text{-}1+}$(B).

Figure S1

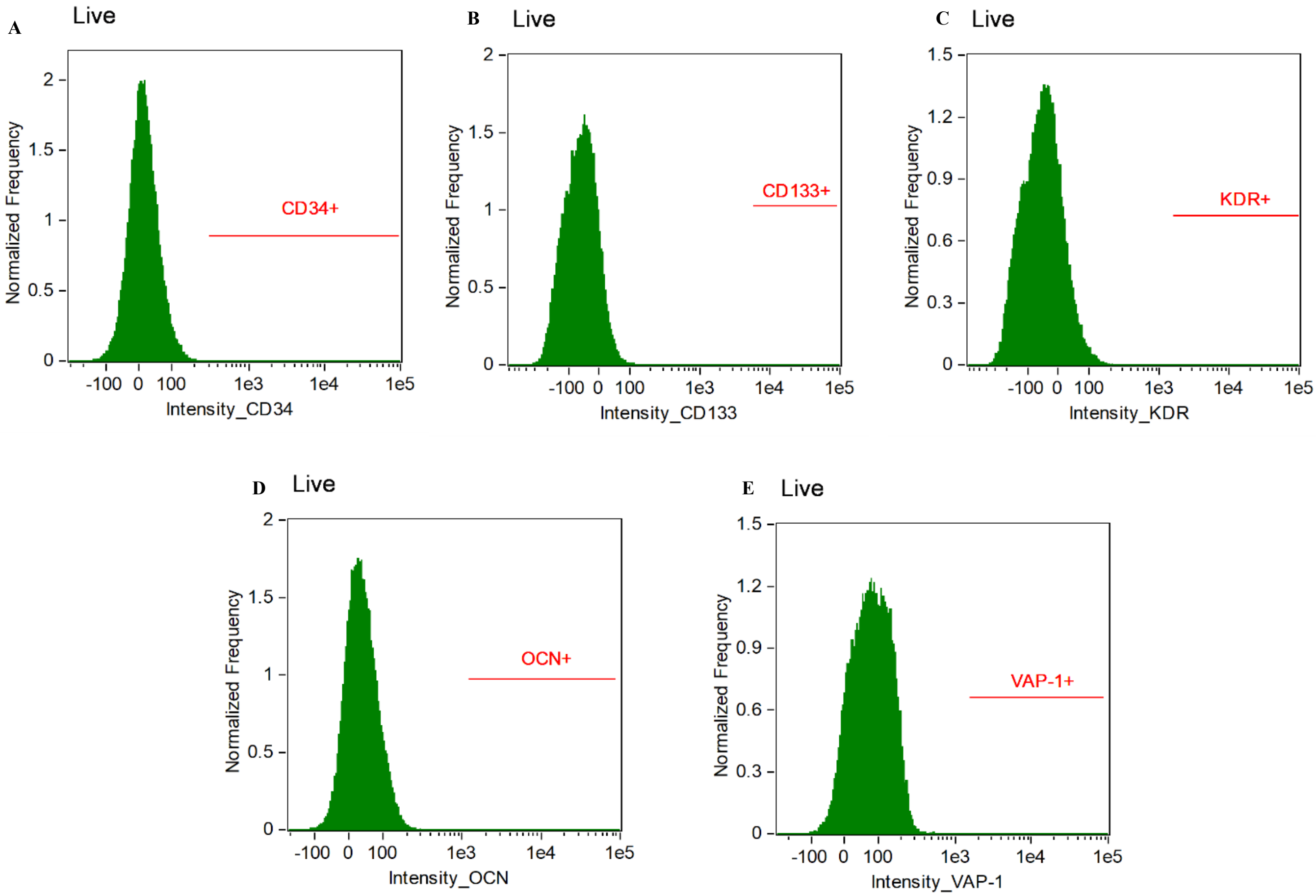

A
Live
Normalized Frequency
2
1.5
1
0.5
0
CD34+
-100 0 100 1e3 1e4 1e5
Intensity_CD34
B
Live
CD133+
Intensity_CD133
C
Live
1.5
1.2
0.9
0.6
0.3
KDR+
Intensity_KDR
D
Live
OCN+
Intensity_OCN
E
Live
VAP-1+
Intensity_VAP-1

Figure S2

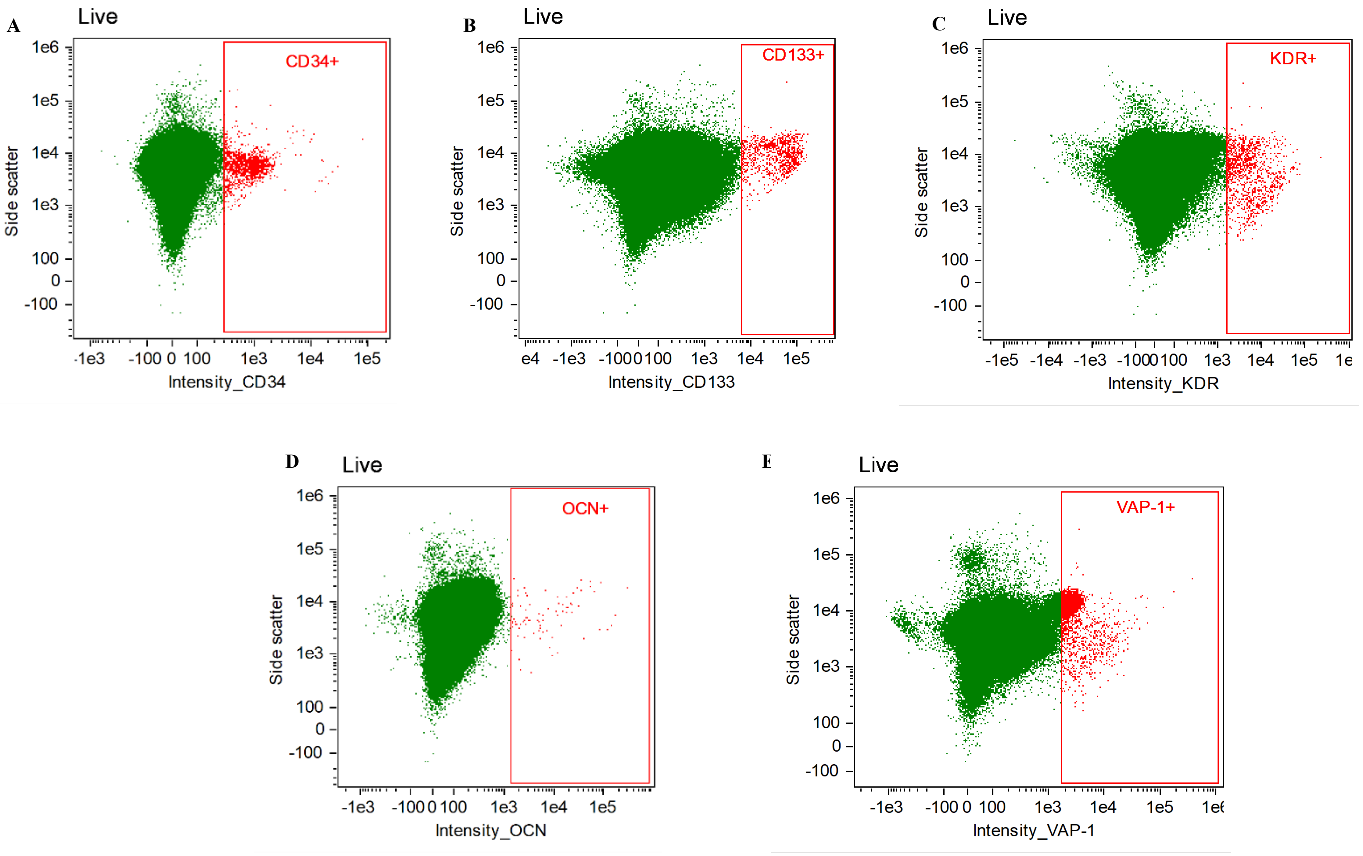
A
Live
CD34+
Side scatter
Intensity_CD34
B
Live
CD133+
Side scatter
Intensity_CD133
C
Live
KDR+
Side scatter
Intensity_KDR
D
Live
OCN+
Side scatter
Intensity_OCN
Live
VAP-1+
Side scatter
Intensity_VAP-1

Figure S3

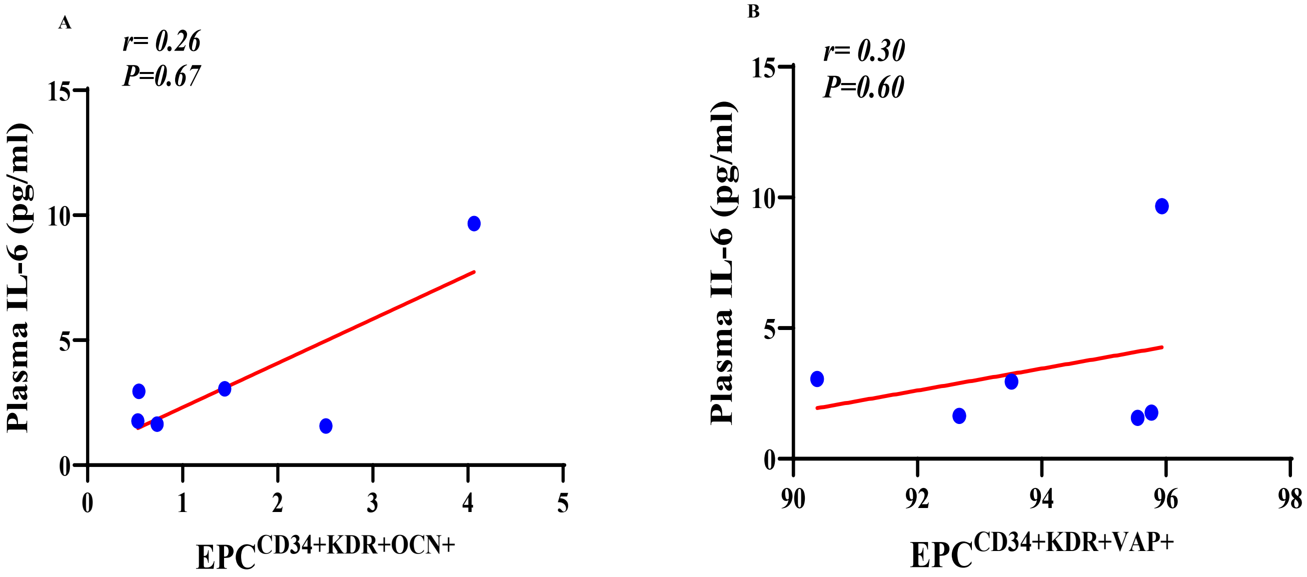

A
r= 0.26
P=0.67
Plasma IL-6 (pg/ml)
15
10
5
0
0
1
2
3
4
5
EPC CD34+KDR+OCN+
B
r= 0.30
P=0.60
Plasma IL-6 (pg/ml)
15
10
5
0
90
92
94
96
98
EPC CD34+KDR+VAP+
